# Experiences With Physical Distancing: Coping Strategies and Positive Experiences

**DOI:** 10.1093/geroni/igab046.1619

**Published:** 2021-12-17

**Authors:** Raven Weaver

**Affiliations:** Washington State University, Pullman, Washington, United States

## Abstract

A representative U.S. sample of adults completed an online survey (N=360) about perceived changes in social health and wellbeing since the implementation of physical distancing restrictions in April. Analyses are conducted on a subsample of adults aged 60+ (n=93; m=65.7 years; SD=4.7). Baseline bivariate descriptive analyses showed no geographic-based differences in self-rated health, resilience, perceived financial wellbeing, or family/friend support measures. Content analysis of rural residents’ (n=20) responses about coping strategies and positive experiences across three time points (April/July/November) revealed aspects of resilience. Individuals coped via acceptance and planning; engaging in activities; and keeping with routines. Positive experiences were relatively stable over time, with individuals describing improved health habits and enhanced social connectedness with family/neighbors. Individuals identified societal betterment and saving money as unintended benefits of the efforts to mitigate the spread of COVID-19. When faced with adversity, identifying positive experiences may help individuals cope with challenges in the long-term

